# Supplementary material for: Plant-Derived Sulforaphane Suppresses Growth and Proliferation of Drug-Sensitive and Drug-Resistant Bladder Cancer Cell Lines In Vitro
Source: Cancers (Basel). 2022 Sep 26;14(19):4682. doi: 10.3390/cancers14194682 (PMC9564120; doi:10.3390/cancers14194682)
Supplement: Supplementary file 1 [file cancers-14-04682-s001.zip › cancers-1896833-supplementary.pdf]

Supplement S1

Cell growth analysis in presence of gemcitabine or cisplatin

Gemcitabine treatment

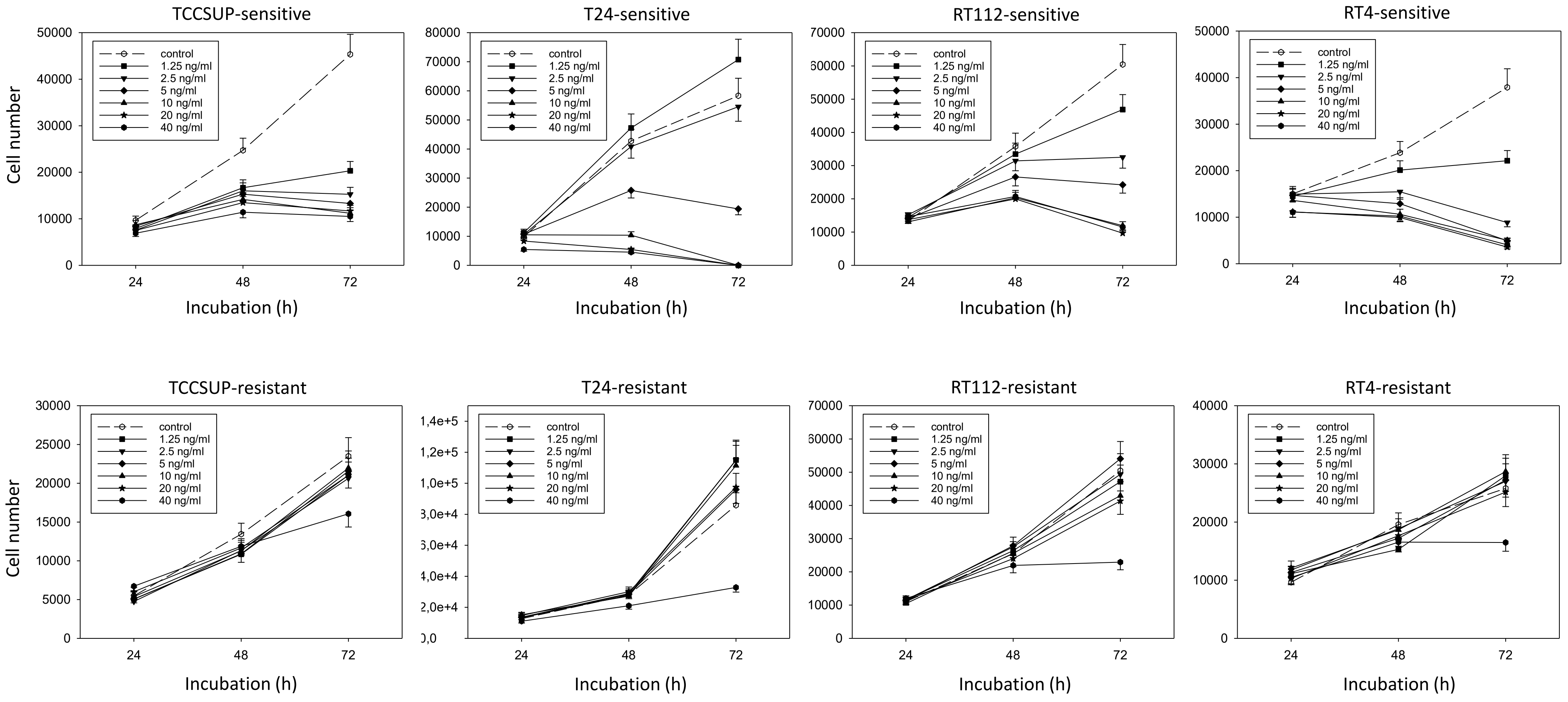

Cisplatin treatment

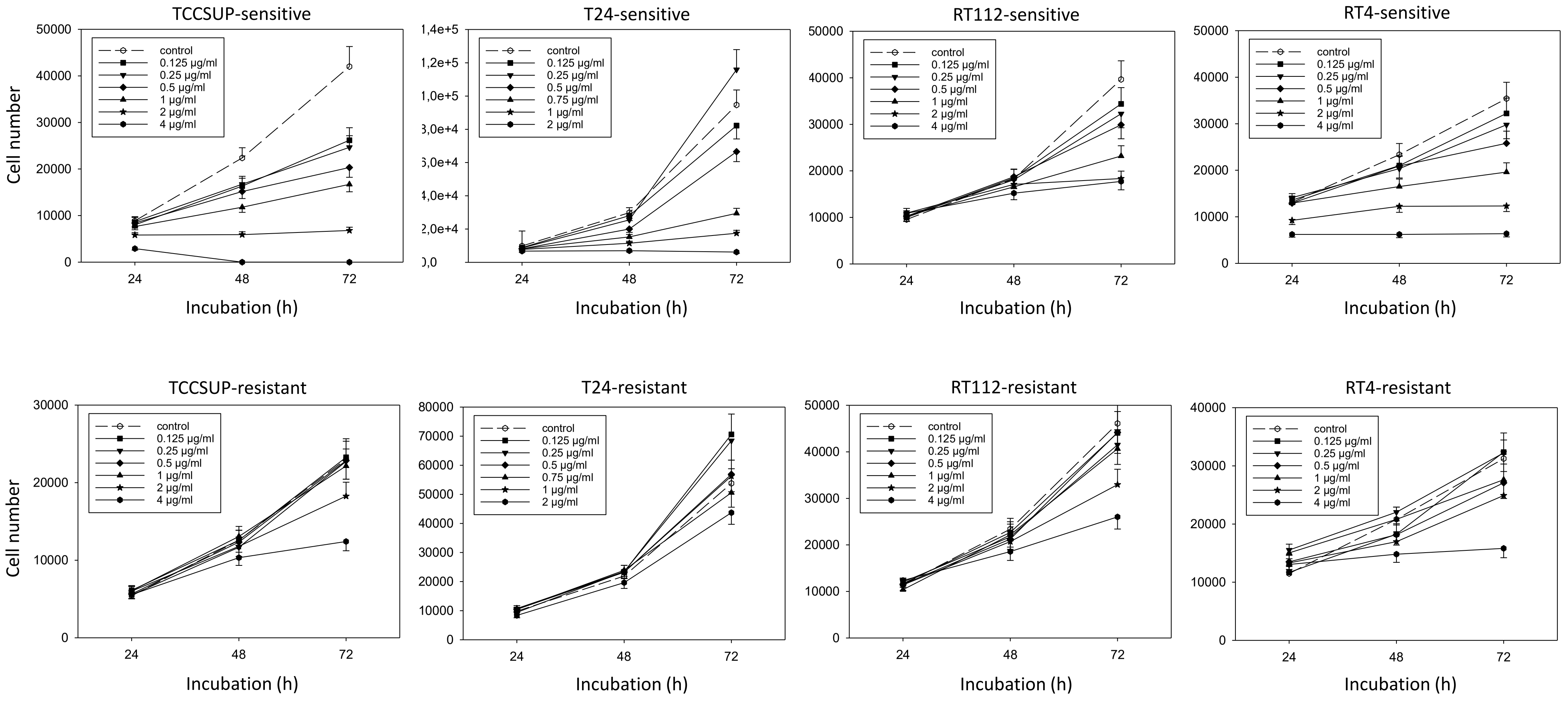

Western blots

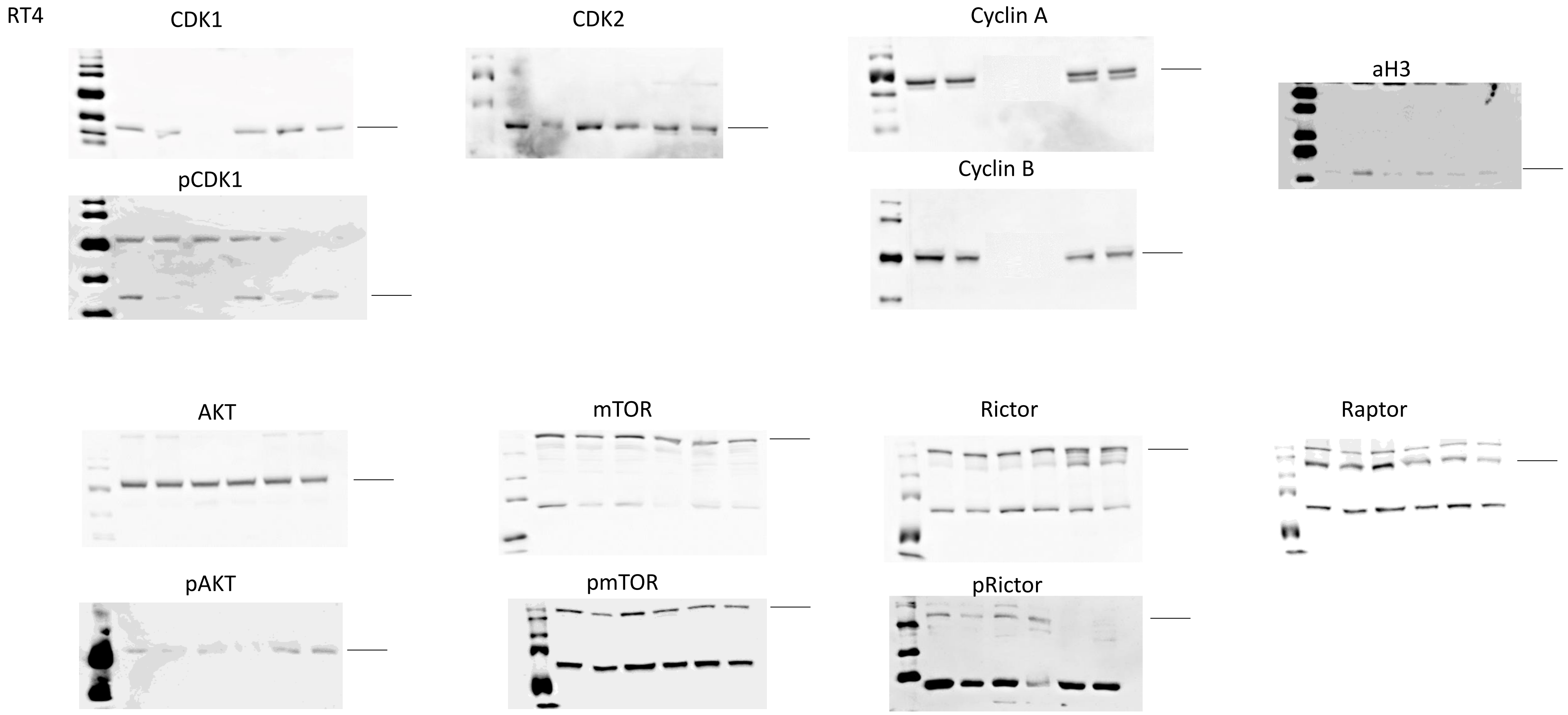

RT112

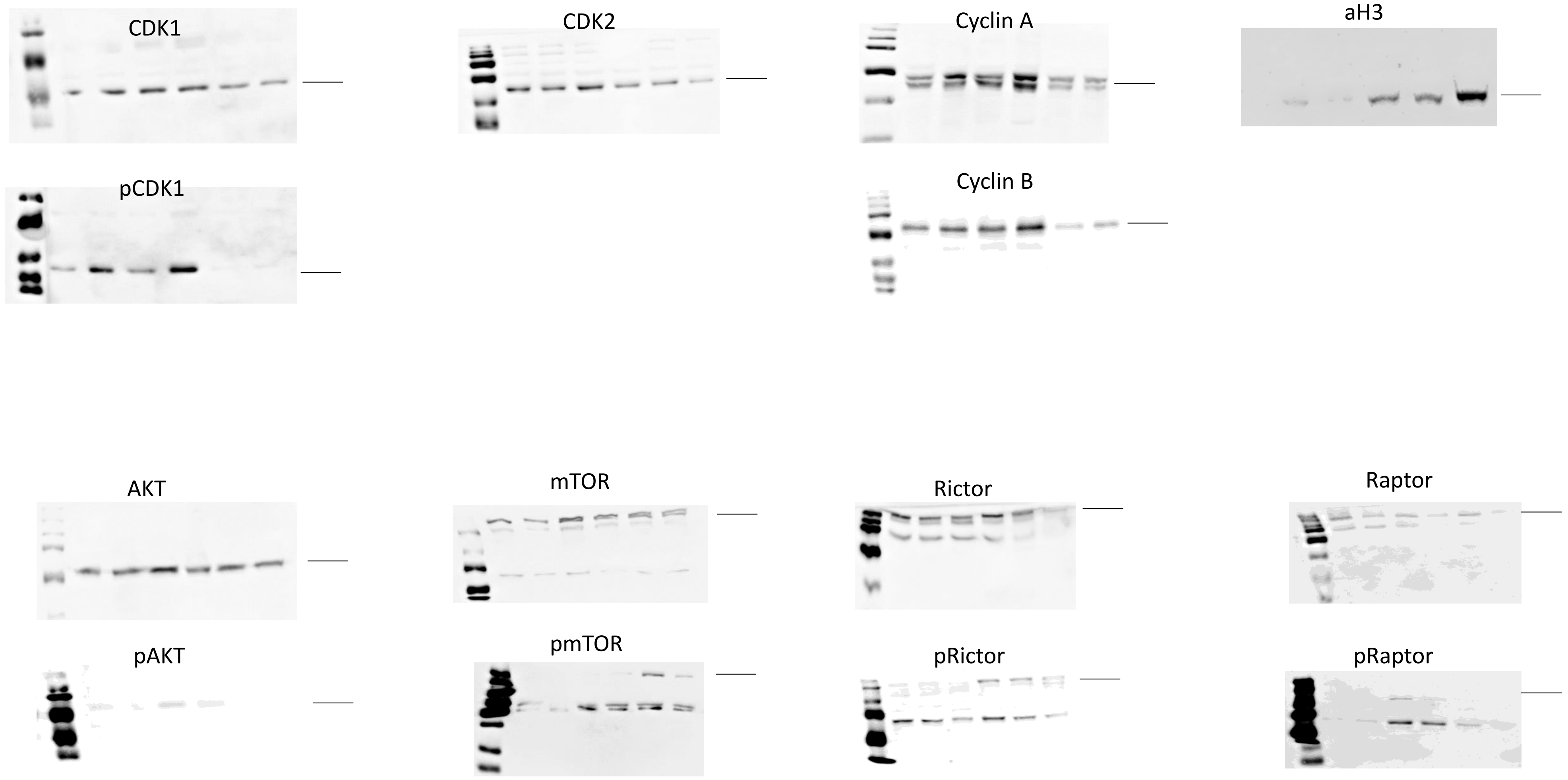

CDK1
pCDK1
CDK2
Cyclin A
Cyclin B
aH3
AKT
pAKT
mTOR
pmTOR
Rictor
pRictor
Raptor
pRaptor

TCCSUP

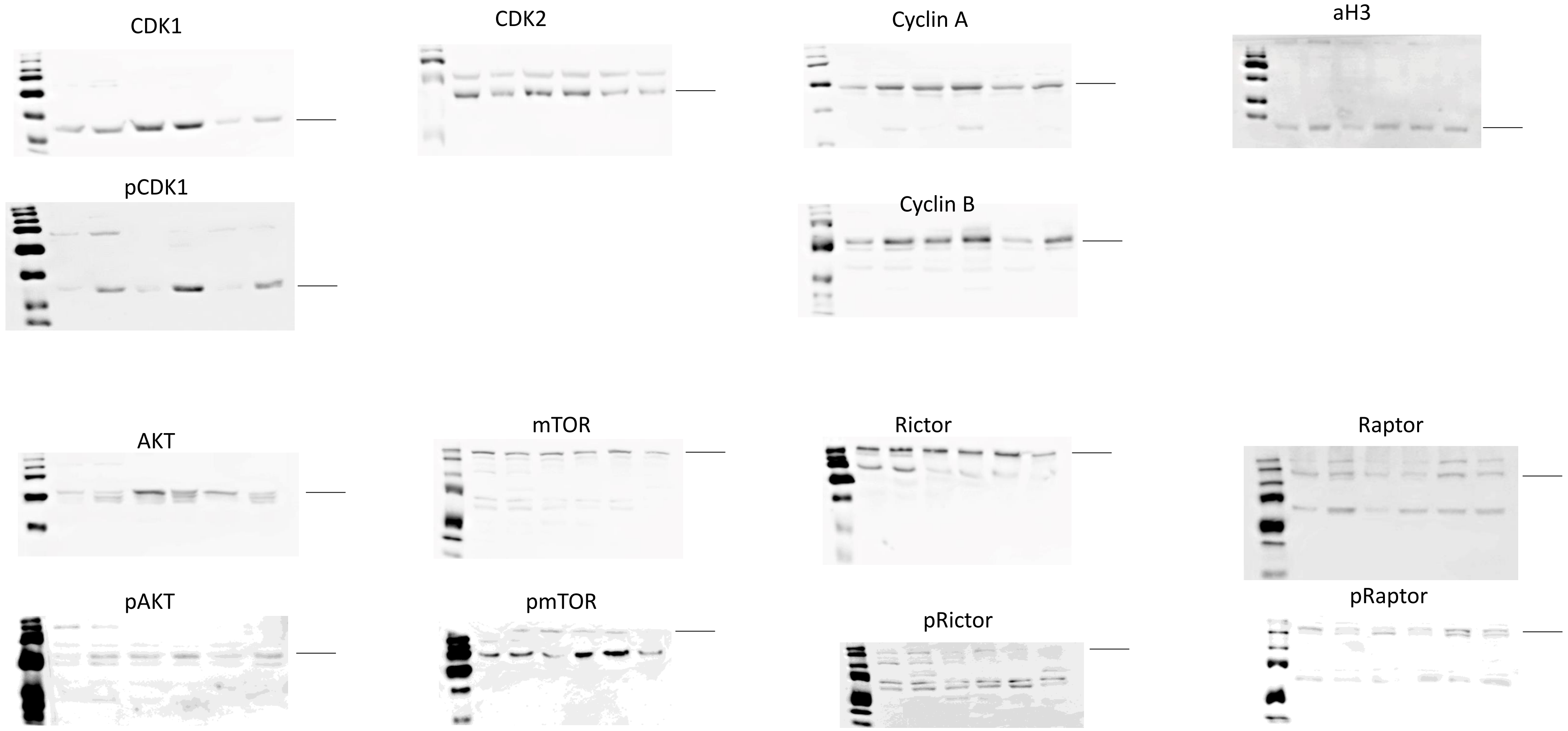
CDK1
CDK2
Cyclin A
aH3
pCDK1
Cyclin B
AKT
mTOR
Rictor
Raptor
pAKT
pmTOR
pRictor
pRaptor

T24

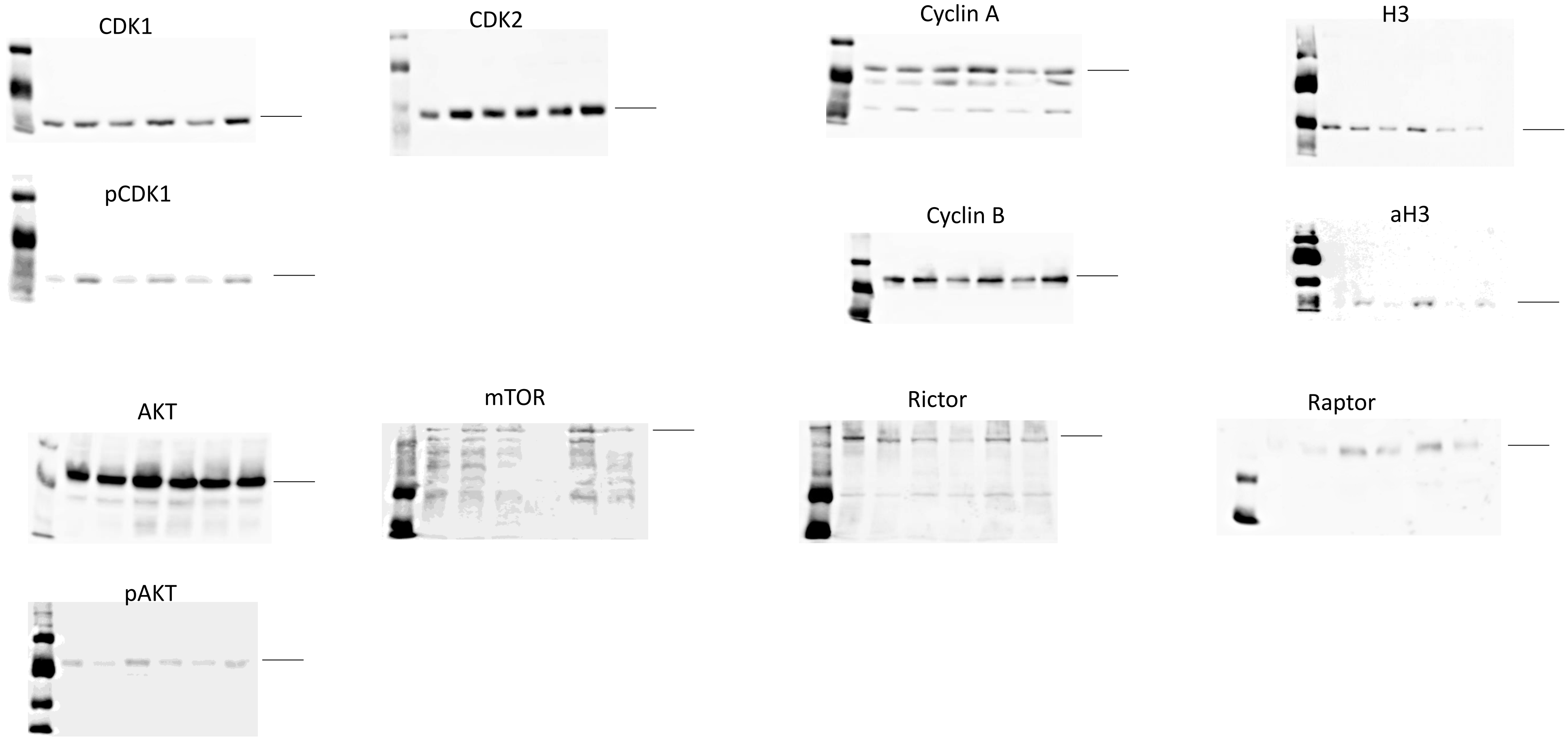
CDK1
CDK2
Cyclin A
H3
pCDK1
Cyclin B
aH3
AKT
mTOR
Rictor
Raptor
pAKT
